# Modelling the effects of 4-factor prothrombin complex concentrate for the management of factor Xa-associated bleeding

**DOI:** 10.1371/journal.pone.0310883

**Published:** 2024-09-27

**Authors:** Ineke Muir, Eva Herzog, Markus Brechmann, Oliver Ghobrial, Alireza Rezvani Sharif, Maureane Hoffman

**Affiliations:** 1 CSL Innovations Pty Ltd, Victoria, Australia; 2 CSL Behring LLC, King of Prussia, PA, United States of America; 3 CSL Behring Innovation GmbH, Marburg, Germany; 4 Department of Pathology, Duke University School of Medicine, Durham, NC, United States of America; The University of Lahore, PAKISTAN

## Abstract

The management of factor Xa (FXa) inhibitor-associated bleeding remains a clinical challenge. Massive bleeding is often associated with complex coagulopathy and, thus, the sole reversal of FXa inhibitors might not be sufficient to restore hemostasis, requiring instead a multimodal approach. Four-factor prothrombin complex concentrate (4F-PCC) is widely recognized as a viable treatment option for FXa inhibitor-associated bleeding. Here, we applied computational models to explore the effect 4F-PCC has on the coagulation cascade and restoration of thrombin generation in a system that simulates a patient that has received a FXa inhibitor. The coagulation model is largely based on a previously developed model with modifications incorporated from various other published sources. The model was calibrated and validated using data from a phase 3 clinical trial of vitamin K antagonist reversal with 4F-PCC. Using the parameters and initial conditions determined during the calibration and validation process, the prothrombin time (PT) test simulations predicted a PT of 11.4 seconds. The model successfully simulated the effects of rivaroxaban and apixaban on total thrombin concentration and showed that 4F-PCC increased thrombin generation in the presence of rivaroxaban or apixaban.

## Introduction

Direct oral anticoagulants (DOACs), including activated factor X (FXa) inhibitors (e.g., apixaban, edoxaban, and rivaroxaban), are increasingly used in clinical practice for the prevention and treatment of thromboembolism [1–3]. However, the use of FXa inhibitors is associated with an increased risk of bleeding. Thus, in situations of life-threatening bleeding or need for urgent surgery, controlling bleeding and restoring hemostasis is vital in FXa inhibitor-treated patients [3,4]. In these cases, a FXa-specific reversal agent, andexanet alfa, is recommended for apixaban and rivaroxaban reversal [5–9]. However, this agent has certain limitations, including high cost, limited availability, complex reconstitution, and safety concerns [2,3]. As a consequence, non-specific agents, such as prothrombin complex concentrate (PCC), are widely recognized as a viable treatment option for FXa inhibitor-associated bleeding, and are thus used by many clinicians [5–14].

**Fig 1:**
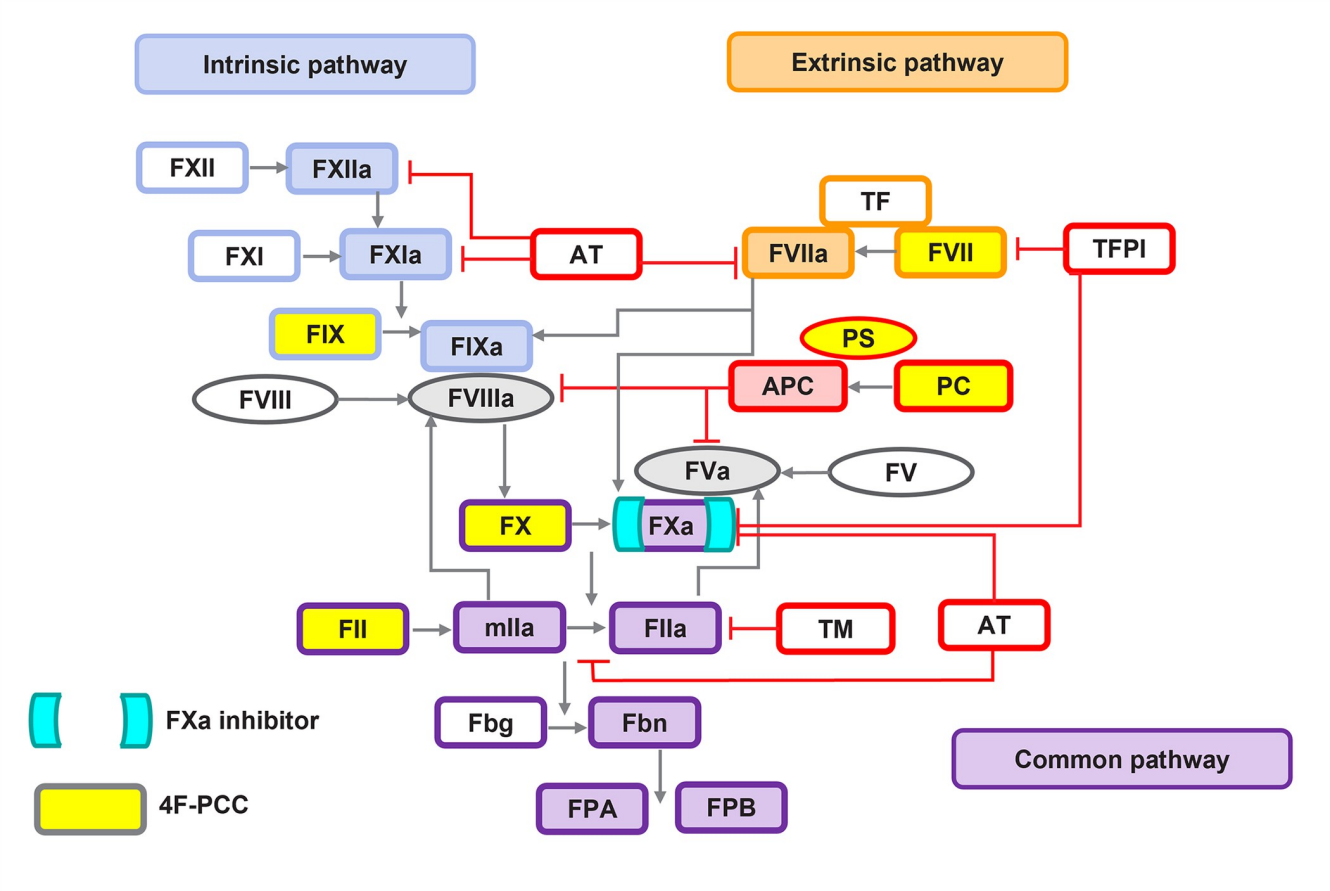
Simplified model reaction network. Factor Xa inhibitor target is shown in aqua brackets and common components in plasma and 4F-PCC formulations in yellow background. Physiological inhibitor reactions are depicted in red. a, activated; APC, activated protein C; AT, antithrombin; F, factor; Fbg, fibrinogen; Fbn, fibrin; FPA, fibrinopeptide A; FPB, fibrinopeptide B; mIIa, meizothrombin; PC, protein C; PS, protein S; TF, tissue factor; TFPI, tissue factor pathway inhibitor; TM, thrombomodulin.

Four-factor PCC (4F-PCC) represents a multimodal therapy which contains non-activated vitamin K-dependent coagulation factors (VKDFs) II, VII, IX, and X (FII, FVII, FIX and FX), and the anticoagulant proteins C and S (PC and PS) [15,16]. A potential advantage of 4F-PCC compared with FXa-specific inhibitors such as andexanet alfa is that its constituents may help treat wider coagulopathy caused by excessive bleeding [4,17]. It has been suggested that by elevating physiological levels of non-activated coagulation factors, 4F-PCC can negate the anticoagulation effects of FXa inhibitors by exerting a prohemostatic effect at the site of injury and increasing thrombin generation, thereby reducing bleeding (see Fig 1) [3,4,17,18]. One of the main components of 4F-PCC is prothrombin (FII), the precursor of thrombin (FIIa), which has been suggested to play a crucial role in the management of bleeding related to FXa inhibitors as well as trauma-associated bleeding [19,20]. However, the exact mechanism of action of individual or combinations of 4F-PCC components is not fully understood and, due to the non-linearity in coagulation system activation, is not able to be explained by simple stoichiometric reactions [17].

Preclinical and clinical studies have evaluated the potential of 4F-PCC for the treatment of FXa inhibitor-associated bleeding. *In vitro* studies have shown that the addition of 4F-PCC to samples of blood with clinically relevant levels of apixaban and rivaroxaban reverses the effects of these drugs on coagulation measurements including thromboelastometry parameters, prothrombin time (PT), and thrombin generation [21,22]. Animal models of bleeding under the effects of FXa inhibitor treatment have shown that 4F-PCC dose-dependently reduced blood loss and improved survival [17,18,23]. These results have been confirmed by multiple clinical studies which have shown that 4F-PCC can effectively restore hemostasis in patients requiring urgent treatment of bleeding associated with FXa inhibitors [24–28].

Clinical studies provide valuable information on outcome measures following treatment with a medication; however, these measures do not tend to directly relate to the mechanism of action of the medication under study, and thus understanding the mechanism of action of medications from clinical data is challenging. *In vitro* assays, such as the thrombin generation assay (TGA) test, are frequently used to gain mechanistic insight [29] into secondary hemostasis but such an approach requires a lot of reagents and time, and may be challenging if the composition of plasma and 4F-PCC need to be modified in many ways, for instance by depletion of individual and combinations of coagulation factors and inhibitors. An alternative approach is to use computational models investigate the mechanisms underlying treatment effects observed in clinical studies [30–32]. Once such a model has been developed and validated, it can be used as a tool to predict the effects of, for example, different dose levels of a therapeutic. In this study, we use a simplified model of the coagulation network that has been calibrated using relevant clinical data sets to explore the effect 4F-PCC has on levels of thrombin generation in a system that simulates a patient who has received a FXa inhibitor [29].

The strength of applying a (well-validated) *in silico* model is the ability to run many simulations, using any number of conditions, to understand how perturbing the input affects a specific output of the coagulation system. On a protein level, coagulation (or secondary hemostasis) is well-characterized and understood; however, the system is highly non-linear and, therefore, it is challenging to predict the effects of changing protein levels.

## Materials and methods

### Model design and validation

The coagulation model is largely based on a model developed by Hockin *et al*. [33] with modifications incorporated from various other published sources [34–36]. Simulations were performed in Matlab R2023b (version 23.2.0.2459199) using the SymBiology application, with ODE solver ode15s, and absolute and relative tolerances of 10^−6^. All reactions are expressed as ordinary differential equations using mass action, with enzymatic reactions expressed as two-step reactions as follows: E + S ES E + P, where E is enzyme, S is substrate and P is product. The model simulates thrombin generation following activation of the coagulation system by tissue factor (TF). It represents a simplified version of the coagulation system and contains all the key pro- and anti-coagulants, as depicted in Fig 1. The table of species with non-zero initial values (and table of all reactions can be found in the Supporting Information.

Reactions occurring at a surface are not captured explicitly; however, the rates of these reactions reflect those that have been measured in the presence of negatively charged lipids and so, the enhanced rates due to these (active) surfaces are encompassed within the model. The model simulates coagulation in a closed system; there is no transport of species into or out of the reaction vessel. Therefore, flow effects are neglected which, in a more physiologically relevant system, would result in dilution of activated species and replenishment of zymogens and inhibitors. Furthermore, the model only considers plasma, but no other blood components such as platelets, which are important in clot formation and stabilization.

The model used here simulates clotting under two conditions. Firstly, it simulates the PT *in vitro* assay and the reaction kinetics for this have been validated against clinical PT test data. Since this assay is carried out in a “closed system” reaction vessel using plasma, the model is able to recapitulate the assay conditions well. In a PT test, very high TF concentrations are used to trigger coagulation, which reduces the sensitivity of the system to minor changes in individual coagulation factor or inhibitor levels. Therefore, for the second condition, clotting is simulated with a more “physiologically” relevant TF level which is orders of magnitude lower than that used for the PT test simulations. This enables a better understanding of the *in vivo* effects on thrombin generation rates due to dosing a FXa inhibitor and/or a 4F-PCC.

### Model validation: Simulation of hemostatic laboratory tests

Fibrin formation occurs very early in the propagation phase of clotting, when only 5–10 nM thrombin has been produced, which is less than 5% of the total thrombin generated during the entire propagation phase [37]. Therefore, it was assumed that the generation of 10 nM thrombin in the simulated PT test corresponds to the formation of fibrin in the assay and signifies the endpoint or clotting time measured in the *in vitro* PT test. Note, thrombin levels in all figures and tables determined via model simulations are the sum of meizothrombin (mIIa) and thrombin (FIIa). Meizothrombin is an intermediate species generated when prothrombin is converted to thrombin by prothrombinase [38].

The concentration of TF used in PT tests is unknown as this information is not provided by the manufacturers. It was assumed that the TF concentration would have been set so as not to be a rate-limiting step in the assay. Therefore, optimization runs were conducted to determine the lowest TF concentration at which the thrombin generation rate was independent of TF concentration, as shown in the Supporting Information. This TF concentration (300 nM) was then used for all subsequent PT simulations.

**Fig 2:**
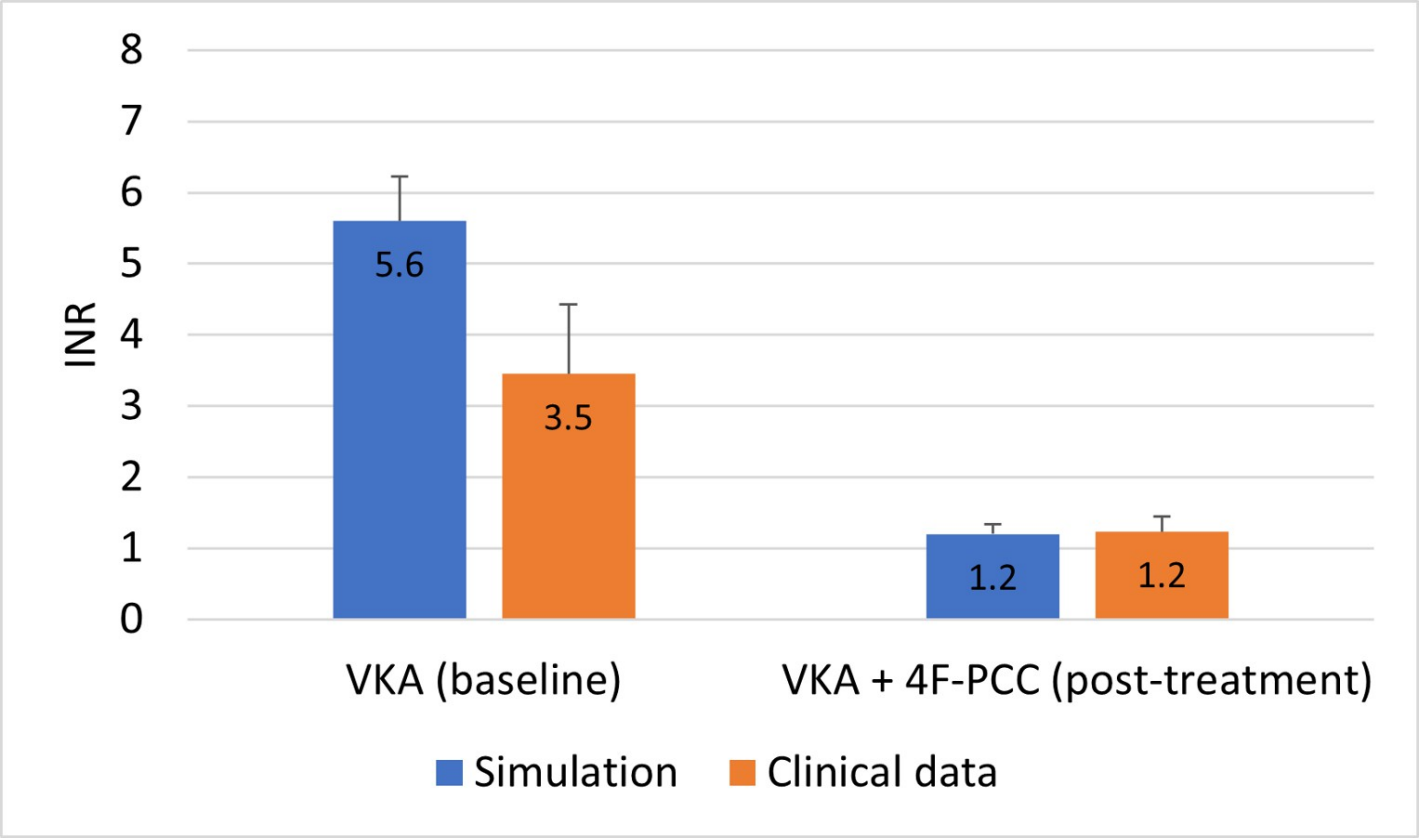
A comparison between the clinical trial prothrombin time test (PT) data (orange) and the simulated PT data (blue), shown are the mean with SD values. 4F-PCC, four-factor prothrombin complex concentrate; INR, international normalized ration; SD, standard deviation; VKA, vitamin K antagonist.

The model was validated using data from a phase 3 clinical trial of vitamin K antagonist (VKA) reversal with 4F-PCC (BE1116_3001, Efficacy and tolerance of Beriplex® P/N in subjects with acquired deficiency of coagulation factors II, VII, IX and X due to oral anticoagulation (CSL Behring, Marburg, Germany) [39]. Seven trial participants’ coagulation factor activity levels on VKA and post-treatment with 4F-PCC were extracted from the study data set as these participants’ data sets were complete and collectively spanned the range from lowest to highest VKDFs prior to 4F-PCC administration. At baseline the average activity of VKDFs II, VII, IX and X in these patients were between 9–36% of normal levels and resulted in an international normalized ratio (INR) ranging from 2.1 to 5.0. After 4F-PCC treatment, these increased to 78–114% of normal factor levels and the subsequent INRs ranged from 1.0 to 1.4. To use these data to validate the model, the factor activity levels pre- and post-treatment were converted to protein concentration and used as inputs to simulate PT times in the model. The concentrations of 4F-PCC components used were the average levels calculated from the ranges specified in the product insert. The specific values are provided in the Supporting Information. A simulated INR is defined as the ratio of the simulated PT time using the patient VKDF levels reported in the clinical trial data to the simulated PT time determined using 100% factor levels. The simulated INRs versus clinical data are shown in Fig 2.

The model was able to accurately reproduce INRs post-4F-PCC treatment (average clinical INR and simulated INR were both 1.2) (Fig 2). The model was less accurate for the pre-treatment INRs, where the discrepancy between the simulation and clinical data for patients on VKA was due to the low factor levels measured in some patients (<10% normal). For average factor levels above 30% of normal, the simulations fit the data well.

To further validate the model beyond the initiation phase of thrombin generation we performed simulations of the TGA, another type of hemostatic screening test that monitors the propagation phase of thrombin generation. This laboratory test uses a much smaller concentration of TF compared with the PT test (between 1 to 5 pM) and a plasma dilution of approximately 40% of physiological concentration (depending on the assay kit) [40]. To check the model responded as expected to the *in vitro* TGA conditions, simulations were performed using 1 and 5 pM TF in 40% physiological plasma protein concentrations. The results correlate well with those expected in a clinical setting using healthy donor blood (see Supporting Information) [41–43]. The model was further validated by comparing simulations of the TGA after the addition of rivaroxaban with published results [44]. The results were in very close agreement to the published data; the addition of 300 ng/mL rivaroxaban resulted in an 88% decrease in the peak thrombin level in the published study and an 87% decrease in the model simulation.

### Ex vivo model

**Fig 3:**
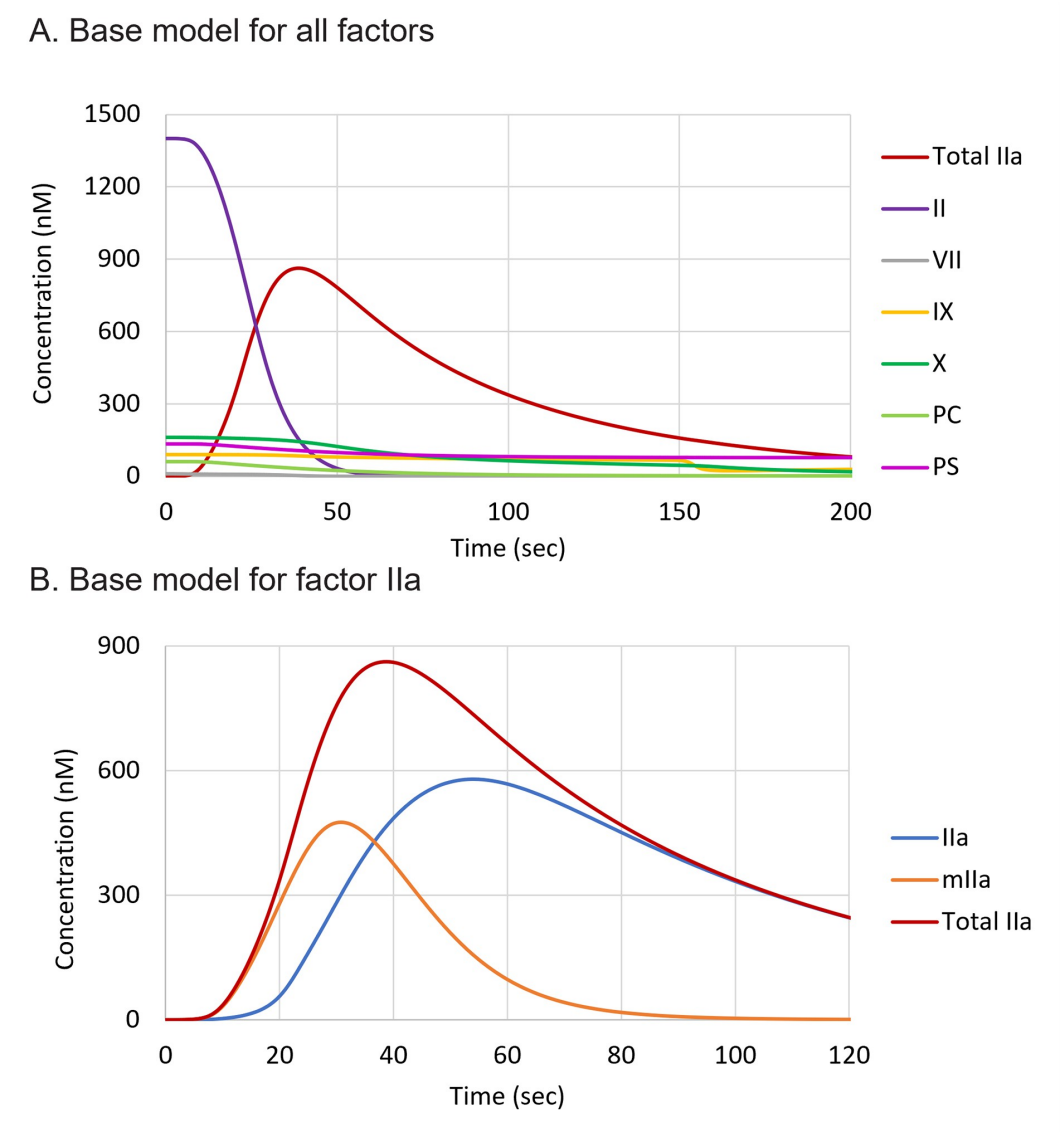
Model results for all factors (A) and for factor IIa (B) using a TF concentration of 4 nM to trigger thrombin generation.

The validated kinetic rates were then used to simulate an “*in vivo”* clotting event using a TF concentration of 4 nM as the trigger (Fig 3), hereafter referred to as the “*ex vivo*” model. It is important to note that in the *ex vivo* case, physiological plasma protein concentrations were used. This differs from *in vitro* plasma-based tests, where the addition of test reagents such as calcium and TF dilute the plasma by varying degrees.

## Results

Using the parameters and initial conditions determined during the validation process, the PT test simulations predicted a PT of 11.4 seconds (Table 1). This PT is within the normal range of 10–13 seconds reported in most laboratories [45]. In the PT assay, the plasma is diluted to one third of its original concentration and a sensitivity analysis indicated that the final TF concentration would be in the vicinity of 300 nM as shown in the Supporting Information. The increase in PT clotting time due to a 20 mg rivaroxaban dose at peak, average, and trough plasma levels were simulated. The effect on PT times due to 4F-PCC at 50, 25, and 15 IU/kg in the presence and absence of rivaroxaban are also shown. PT simulations for apixaban were not conducted due to ICSH guidelines reporting that the PT test is not sensitive to apixaban levels in patient plasma [46].

**Table 1:** Clotting times for the simulations of the PT test.

		PT endpoint (sec)
	**Base model**	11.4
	**Rivaroxaban**	
	Peak	45.7
	Average	22.6
	Trough	12.1
	**4F-PCC (IU/kg)**	
	50	10.0 9.3 8.2
	25	
	15	
**4F-PCC (IU/kg)**	**Rivaroxaban**	
50	Peak	25.1
	Average	13.8
	Trough	8.6
25	Peak	31.5
	Average	16.6
	Trough	9.7
15	Peak	35.8
	Average	18.5
	Trough	10.3

Rivaroxaban levels are the plasma concentrations following a 20 mg daily dose; peak, average, and trough plasma levels are 282, 83, and 4 ng/mL respectively.4F-PCC, four-factor prothrombin complex concentrate; PT, prothrombin time; IU, international unit.

In Fig 3 is shown the consumption of coagulation factors following a trigger of 4 nM TF (*ex vivo* model), due to conversion of the zymogens to their active, enzymatic forms and subsequent inhibition by endogenous inhibitors such as tissue factor pathway inhibitor (TFPI) and antithrombin (AT). Since the model does not include transport of species into and out of the reaction space (closed system), most of the prothrombin is rapidly converted to thrombin and mIIa.

### Simulation of FXa inhibitors

The pharmacokinetic parameters of FXa inhibitors rivaroxaban and apixaban were used to determine the peak, average and trough plasma levels between doses [47,48], which were used in the model simulations. The highest recommended doses were chosen for both FXa inhibitors to simulate the most extreme cases; this corresponded to 20 mg per day for rivaroxaban and 10 mg twice daily for apixaban (note this is the 7-day loading dose for deep vein thrombosis and pulmonary embolism). We chose the higher dose of apixaban in the loading phase as it has been shown that the risk of bleeding in patients (with atrial fibrillation) correlated with anit-FXa levels and hence, plasma FXa inhibitor levels [49]. Since rivaroxaban does not have a separate loading dose, we used the maintenance dose.

**Fig 4:**
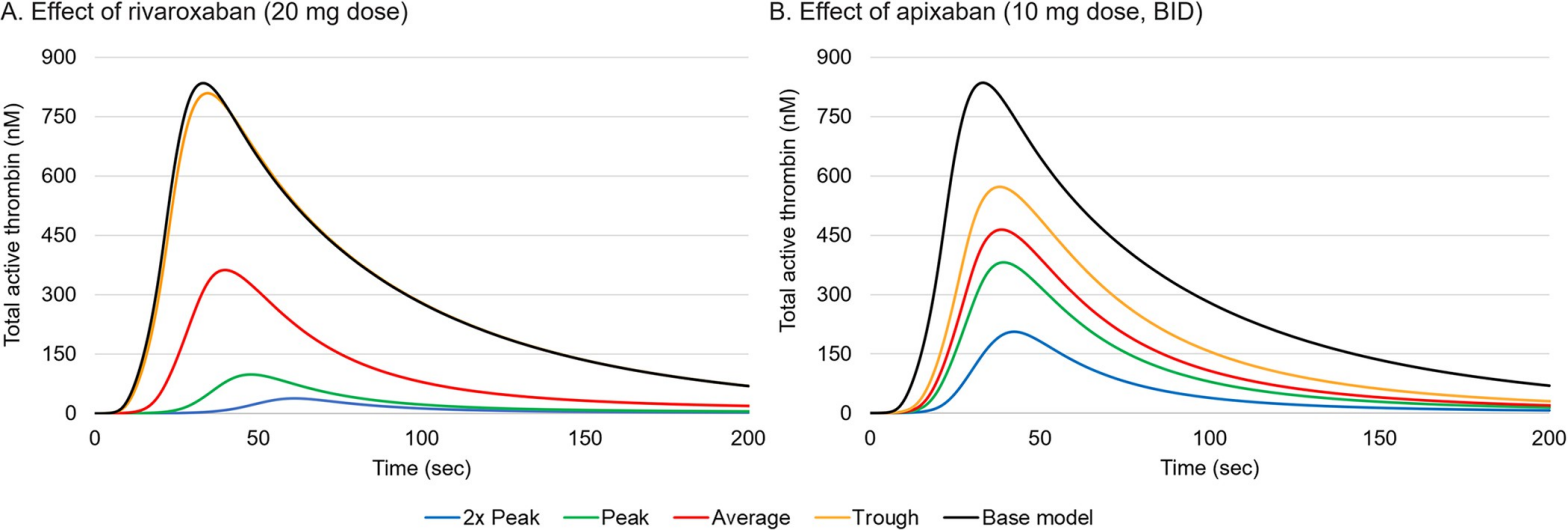
Simulation of the effect of rivaroxaban (A) and apixaban (B) using a TF concentration of 4 nM. BID, twice a day.

Fig 4A shows the effect of a 20 mg dose of rivaroxaban on thrombin generation using the *ex vivo* model. Peak plasma levels for a 20 mg dose corresponded to a rivaroxaban concentration of 282 μg/L [47]. At trough levels the concentration of rivaroxaban was much lower at 4 μg/L and the simulated peak thrombin is close to that without any FXa inhibitor. The average concentration of rivaroxaban was 83 μg/L. Fig 4B shows the effect of a twice daily 10 mg dose of apixaban on total FIIa concentration. At peak plasma levels, occurring twice every 24 hours, the concentration of apixaban was 251 μg/L [48], and at trough levels the FXa inhibitor is at 120 μg/L, which is still relatively high (compared with the trough rivaroxaban level), resulting in a 30% reduction in peak thrombin compared with the base case. The average concentration of apixaban was 185 μg/L.

The proportion of FXa inhibitor bound to FXa for both rivaroxaban and apixaban at peak and trough levels is shown in S2 Fig in S1 File. At the peak level of rivaroxaban, only around 20% of the total plasma levels of inhibitor is bound to FXa whereas at the trough level all the inhibitor is bound. For apixaban, the difference between the proportion bound to FXa at peak and trough levels is less extreme; at peak apixaban, just over 20% is bound and at the trough levels 55% is bound.

### Simulation of 4F-PCC

The addition of 4F-PCC in the presence of either rivaroxaban or apixaban influences the simulated thrombin generation. Table 1 shows the effect on PT test simulations of rivaroxaban and 4F-PCC, both separately and in combination. Note that apixaban was excluded from these simulations since it has been reported that this laboratory test is not sensitive to plasma apixaban levels [46,50,51]. As expected, the extent of the effect depends on the relative levels of FXa inhibitor and 4F-PCC. In all cases, 4F-PCC increased thrombin levels and decreased PT relative to FXa inhibitors alone. At peak plasma levels of rivaroxaban, the PTs were reduced from 45.7 seconds in the absence of 4F-PCC to 25.1, 31.5, and 35.8 seconds for 4F-PCC doses of 50, 25, and 15 IU/kg, respectively. At average rivaroxaban levels, the PTs were reduced from 22.6 to 13.8, 16.6, and 18.5 seconds for 50, 25, and 15 IU/kg 4F-PCC, respectively. And finally, at trough rivaroxaban levels, 50, 25, and 15 IU/kg 4F-PCC reduced the PT from 12.1 to 8.6, 9.7, and 10.3 seconds respectively.

**Fig 5:**
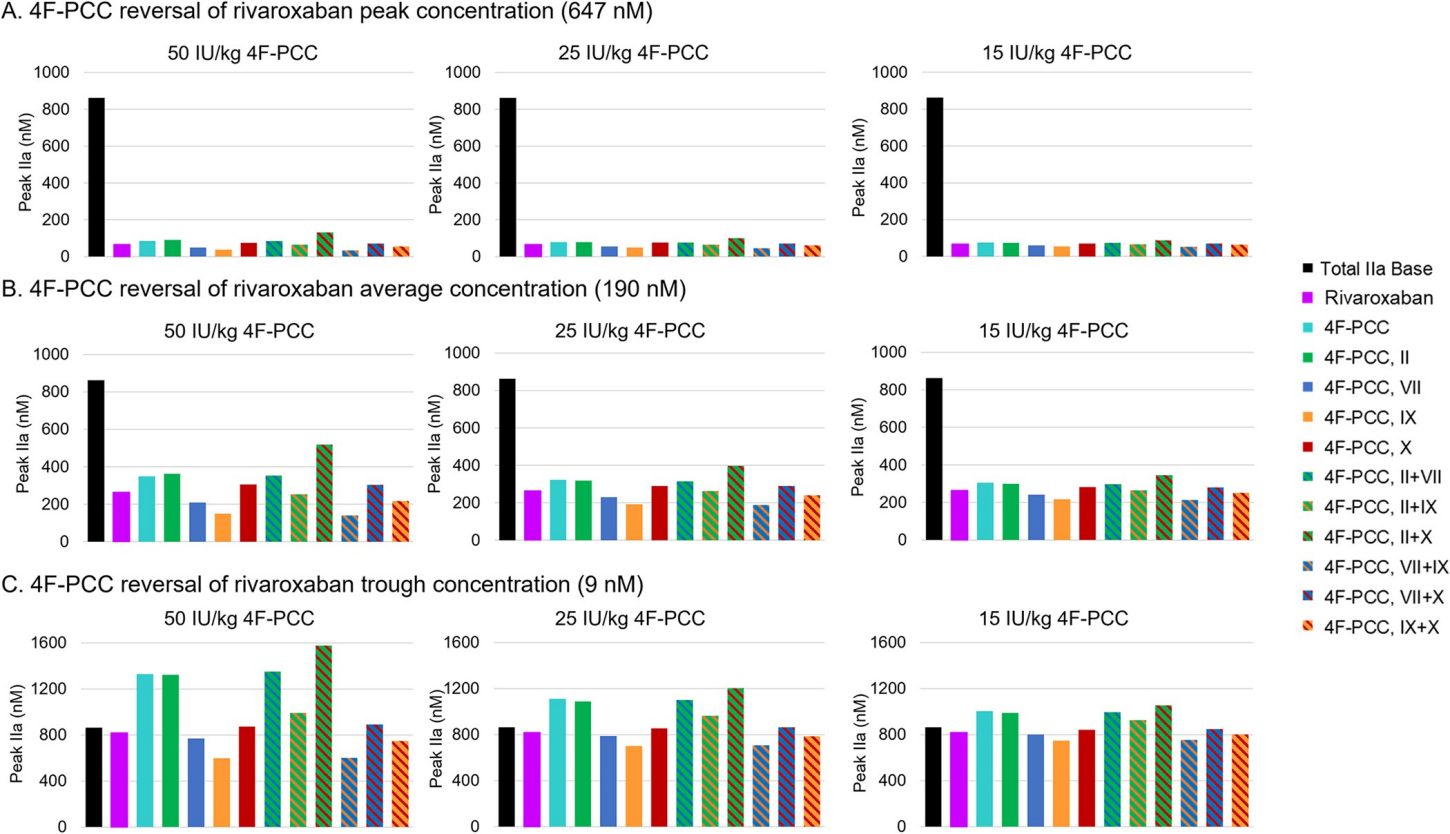
Rivaroxaban reversal with 4F-PCC (50 IU/kg, 25 IU/kg, and 15 IU/kg), using a TF concentration of 4 nM. The bars in each plot represent simulations of total IIa in the presence of rivaroxaban and 4F-PCC (all components), individual pro-coagulant components of 4F-PCC (FII, FVII, FIX, FX), and different combinations of two 4F-PCC components (FII and FVII, FII and FIX, FII and FX, FVII and FIX, FVII and FX, FIX and FX) compared with the base model total IIa results. The inhibitors PC and PS were included at the appropriate level in all the simulations. 4F-PCC, four-factor prothrombin complex concentrate; FII, factor II, FVII, factor VII; FIX, factor IX; FX, factor X; IU, international unit; PC, protein C; PS, protein S; TF, tissue factor.

For the *ex vivo* case, at peak rivaroxaban, there was only a minor increase in thrombin generation even at the highest dose of 4F-PCC. At average rivaroxaban levels, the highest 4F-PCC dose (50 IU/kg) was not able to restore thrombin levels equal to the base case (in the absence of a FXa inhibitor). At trough levels, all three doses of 4F-PCC (50, 25, and 15 IU/kg) were able to fully restore thrombin levels (Fig 5) compared with the untreated case. The trends were similar for the *ex vivo* simulations with apixaban; however, the effects on thrombin generation due to the FXa inhibitor on its own were not as profound, due to the flatter peak and trough concentrations resulting from a twice-daily dose compared with the daily rivaroxaban dosing regimen. 4F-PCC at all doses (50, 25, and 15 IU/kg) increased thrombin generation compared with apixaban alone, but was unable to restore thrombin to pre-FXa inhibitor levels in any of the simulations.

**Fig 6:**
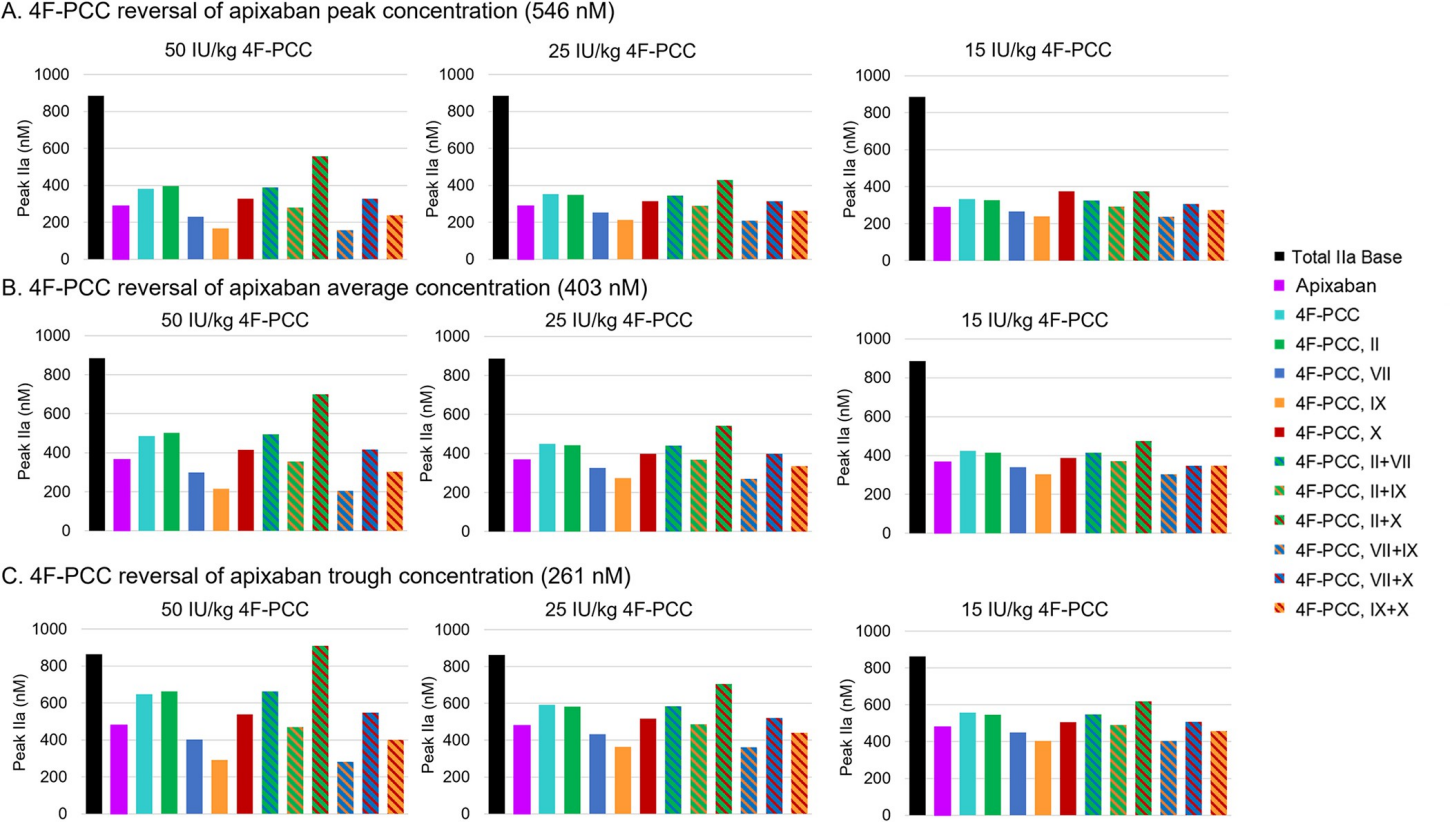
Apixaban reversal with 4F-PCC (50 IU/kg, 25 IU/kg, and 15 IU/kg) using a TF concentration of 4 nM. The bars in each plot represent simulations of total IIa in the presence of apixaban and 4F-PCC (all components), individual pro-coagulant components of 4F-PCC (FII, FVII, FIX, FX), and different combinations of two 4F-PCC components (FII and FVII, FII and FIX, FII and FX, FVII and FIX, FVII and FX, FIX and FX) compared with the base model total IIa results. The inhibitors PC and PS were included at the appropriate level in all the simulations. 4F-PCC, four-factor prothrombin complex concentrate; FII, factor II, FVII, factor VII; FIX, factor IX; FX, factor X; IU, international unit; PC, protein C; PS, protein S; TF, tissue factor.

The effects of the individual components of 4F-PCC at rivaroxaban and apixaban peak, trough, and average levels on FIIa are presented in Figs 5 and 6 for the different doses of 4F-PCC. The trends were very similar across all rivaroxaban and apixaban levels and 4F-PCC component levels, and the magnitude of the effect on thrombin generation scaled with dose levels. Prothrombin alone (at the concentrations equivalent to that in the 4F-PCC doses indicated) increased FIIa concentration to approximately the same levels as the complete 4F-PCC formulation and had a greater effect on FIIa than any of the other individual components. Note, PC and PS were included in all simulations. FIX alone (again at the levels present in the 4F-PCC) had the least effect on restoring the FIIa levels, followed by FVII. The combination of FII and FX increased FIIa concentration the most compared with 4F-PCC, the other individual factors, and combinations of factors, followed by the combinations of FII with FVII, and FVII with FX.

## Discussion

Here, we applied a computational modelling approach to explore the effect of 4F-PCC on the coagulation network and restoration of thrombin generation in a system that simulates a patient who has received a FXa inhibitor. A computational “*in vivo*” model was generated, termed the “*ex vivo*” model which consisted of all the coagulation factors, cofactors and inhibitors listed in the Supporting Information, at *in vivo* levels. Coagulation was triggered in the model via the addition of TF.

There is some uncertainty as to what constitutes a suitable TF concentration for this purpose. Several literature sources have measured soluble TF levels in healthy donor blood and report systemic levels in the pM range, which, under conditions of normal blood flow, is too low to cause robust coagulation. However, true TF levels are difficult to quantify as TF is a transmembrane protein and so the majority of it is unmeasurable as a soluble biomarker in blood. TF levels can also vary considerably, depending on the disease state and physiological location. For example, TF expression in monocytes and endothelial cells occurs predominantly during inflammation or thrombogenesis [52] and pathological conditions such as sepsis, atherosclerosis and cancer can result in aberrant TF expression within the vasculature and cause thrombotic complications [53]. Immunohistochemical studies have shown high expression of TF in healthy sub-endothelial tissue, both on the surface and within extravascular cells such as fibroblasts, pericytes and epithelial cells which would be exposed if the blood vessel walls were injured [54]. The aim of this study was to understand the mechanisms occurring when 4F-PCC is used to reverse the effects of FXa inhibitors. Clinically, this scenario would only be necessary in the event of heavy bleeding. Under such circumstances, there would be significant damage to the blood vessel wall, potentially exposing large amounts of TF. To mimic this, a TF concentration of 4 nM was chosen, as this produced robust and rapid thrombin generation in the model. It has been reported that very high TF levels reduce the sensitivity of clotting tests to the effects of FXa inhibitors [55]. However, in the *ex vivo* model, 4 nM TF was sufficiently low such that the amount and rate of thrombin generation were sensitive to the effects of changes in FXa inhibitor and 4F-PCC levels, as shown in Figs 4–6.

The model presented in this study successfully simulated the effects different 4F-PCC doses have on a patient treated with apixaban or rivaroxaban. The results showed that 4F-PCC at all dose levels reduced the PT relative to that in the presence of rivaroxaban alone. At peak rivaroxaban concentration, the PT was reduced from 45.7 s to 25.1 s with 50 IU/kg rivaroxaban and to 35.8 s with 15 IU/kg. At trough rivaroxaban concentration, the PT was already within normal range (12.1 s) and the addition of 4F-PCC, even at the highest dose of 50 IU/kg, only had a minor effect of reducing this to 8.6 s. The levels of FIIa required to ensure hemostasis is restored are unknown, so it cannot be ascertained whether the simulated reduction in PTs due to the addition of 4F-PCC are sufficient to have a clinical effect. However, it is not unlikely that the simulations presented here underestimate the level of thrombin generated since 4F-PCC also increases platelet recruitment [21], an effect that is not accounted for in the model since only plasma was considered.

When the effects of the different components of 4F-PCC individually were simulated, we observed that prothrombin increased thrombin levels the most compared with a complete 4F-PCC formulation and its other individual components. When combinations of factors are considered together, the model predicted that FII plus FX generate the highest thrombin concentration, similar to the levels achieved from the complete 4F-PCC, and greater than the other individual factors alone. This corroborates the findings of Eltringham-Smith *et al*. who concluded that FII was the dominant procoagulant component of PCC in a reductionist coagulopathic mouse model [19]. The fact that FII is the most important procoagulant component suggests there is redundancy in physiological concentrations of enzymes upstream of FIIa formation, as evidenced from *in vivo* data where in a model of depleted coagulation factors (20% of normal) restoration of hemostasis comparable to that by 4F-PCC was observed after administration of high levels of FII alone [19]. This indicates that, even in the presence of only 20% normal prothrombinase complex concentration, if there are high levels of FII available, sufficient FIIa will be formed. Presumably, this is because the high levels of FII, combined with the increase in local levels of prothrombinase complex (which acts as a recyclable catalyst) in the vicinity of the wound are able to drive efficient conversion of FII to FIIa. At the same time, this also has implications in clot stability, since FIIa converts fibrinogen to fibrin, and thus concentrates fibrin in the region of the clot. The fibrinogen that is converted to fibrin would also contain bound platelets, thereby further stabilizing the clot. This has also been evidenced *in vivo* where an increase in platelets was measured following PCC and FII dosing in a mouse model of blood exchange-induced coagulopathy [19].

In Fig 6, at peak apixaban concentration, the addition of FX alone at 50 and 25 IU/kg generated less thrombin than FII alone, whereas at 15 IU/kg, FX had the greater impact. Possibly this is an effect from the concomitant reduced PC and PS that inhibit FVIIIa and FVa and, therefore, less prothrombinase is produced, resulting in a decrease in FII activation. This observation is an example of the non-linear behavior of the system and the challenges faced when trying to predict the outcome of a complex system based on the perturbation of a single input; it also demonstrates the insight that can be gained from mechanistic modeling.

When simulating binary mixtures of 4F-PCC components, in the presence of the appropriate PC and PS levels, the simulations indicated that FII with FX was the most effective combination across all FXa inhibitor and 4F-PCC component levels, and the magnitude of the effect scaled with concentration of the FXa inhibitor. Indeed, the combination of FII and FX generated more thrombin than the complete 4F-PCC formulation and this observation fits with the hypothesis that FII is the most important component for thrombin generation. The additional presence of FX would add to the systemic FX levels and serve to reduce the effect of the FXa inhibitor, thereby allowing more prothrombinase complex to form and hence increasing thrombin levels.

It is interesting to consider the impact that the vitamin K-dependent inhibitors, PC and PS, have on the mechanism of action of 4F-PCC. PC and PS directly inhibit cofactors FV and FVIII. FVIII is required to accelerate FIX-mediated activation of FX and FV is required for the formation of the prothrombinase complex. To explore this, we generated simulations of the individual and binary mixtures of 4F-PCC at 50 IU/kg, with and without the inhibitors, at average rivaroxaban and apixaban levels, shown in the Supporting Information. For both FXa inhibitors, the presence of PC and PS reduced thrombin generation in all cases with the exception of when just FX, FII with FX, and FVII with FX were added. The common 4F-PCC component here is FX, and the insensitivity of the system to PC and PS in the presence of additional FX may suggest that a lack of uninhibited FX is a key rate-limiting step in thrombin generation. The addition of extra FX compensates for both the inhibitory effect of the DOACs as well as the inhibition due to PC and PS on catalytic efficiency of the cofactors.

The management of FXa inhibitor-associated bleeding remains a clinical challenge. Massive bleeding is often associated with complex coagulopathy and thus, the sole reversal of FXa inhibitors might not be sufficient to restore hemostasis, requiring instead a multimodal approach, particularly following trauma [4,11]. Moreover, thrombin generation may be altered not only by FXa inhibitor use, but also by dilution and consumption of coagulation factors [11,56]. Here we showed that the concentration of FX in 4F-PCC was not sufficient to overcome the effects of the FXa inhibitor. Since the half-life of FX is much longer than that of a FXa inhibitor [57], dosing sufficient levels of additional FX to overcome the inhibition by a FXa inhibitor would not be appropriate, especially at peak FXa inhibitor levels (see figure in the Supporting Information section showing that there is excess FXa inhibitor levels relative to FXa), since the exogenous FX will persist in the system far longer than the FXa inhibitor, and therefore, significantly elevate total FX levels. Our model also showed that FII in combination with either FIX, FVII, and to a lesser extent FX, effectively increased thrombin concentration. Accordingly, in a recent *in vivo* pig polytrauma model, which included dilution and consumption of factors, 4F-PCC was shown to be effective at reducing blood loss and restoring thrombin generation in animals anticoagulated with rivaroxaban [17]. Similar to our results, the omission of FII from 4F-PCC has been shown *in vivo* to significantly limit its efficiency in reducing bleeding, suggesting that prothrombin in 4F-PCC has a key role [19].

## Conclusions

Computational models can be used to simulate treatment effects of 4F-PCC observed in clinical studies of FXa inhibitor-associated bleeding and to probe the mechanism of FXa inhibitor reversal via the complete 4F-PCC formulation. Such models can also be used to probe the mechanism of action by determination of the quantitative contribution to thrombin generation by individual components in 4F-PCC as well as combinations of components. Our model showed that 4F-PCC effectively increased thrombin generation. Of particular interest, prothrombin alone was shown to be the most crucial component for reversing the effect of the FXa inhibitors and restoring thrombin. Prothrombin in combination with FX was the most effective at generating thrombin compared with prothrombin alone or in combination with FVII. FIX had the least effect on thrombin generation, whether added alone or in combination with other 4F-PCC components.

## Supporting information

S1 File Contains supporting tables and figures. (DOCX)

## References

[1] Weitz JI, Eikelboom JW, Samama MM. New antithrombotic drugs: Antithrombotic Therapy and Prevention of Thrombosis, 9th ed: American College of Chest Physicians Evidence-Based Clinical Practice Guidelines. Chest. 2012;141(2 Suppl):e120S–e51S. doi: 10.1378/chest.11-2294 22315258 PMC3278067

[2] Kustos SA, Fasinu PS. Direct-Acting Oral Anticoagulants and Their Reversal Agents-An Update. Medicines (Basel). 2019;6(4). doi: 10.3390/medicines6040103 31618893 PMC6963825

[3] Grottke O, Schulman S. Four-factor Prothrombin Complex Concentrate for the Management of Patients Receiving Direct Oral Activated Factor X Inhibitors. Anesthesiology. 2019;131(5):1153–65. doi: 10.1097/ALN.0000000000002910 31415251

[4] Hoffman M, Goldstein JN, Levy JH. The impact of prothrombin complex concentrates when treating DOAC-associated bleeding: a review. Int J Emerg Med. 2018;11(1):55. doi: 10.1186/s12245-018-0215-6 31179943 PMC6326120

[5] Witt DM, Nieuwlaat R, Clark NP, Ansell J, Holbrook A, Skov J, et al. American Society of Hematology 2018 guidelines for management of venous thromboembolism: optimal management of anticoagulation therapy. Blood Adv. 2018;2(22):3257–91. doi: 10.1182/bloodadvances.2018024893 30482765 PMC6258922

[6] Tomaselli GF, Mahaffey KW, Cuker A, Dobesh PP, Doherty JU, Eikelboom JW, et al. 2020 ACC Expert Consensus Decision Pathway on Management of Bleeding in Patients on Oral Anticoagulants: A Report of the American College of Cardiology Solution Set Oversight Committee. J Am Coll Cardiol. 2020;76(5):594–622. doi: 10.1016/j.jacc.2020.04.053 32680646

[7] Baugh CW, Levine M, Cornutt D, Wilson JW, Kwun R, Mahan CE, et al. Anticoagulant Reversal Strategies in the Emergency Department Setting: Recommendations of a Multidisciplinary Expert Panel. Ann Emerg Med. 2020;76(4):470–85. doi: 10.1016/j.annemergmed.2019.09.001 31732375 PMC7393606

[8] Cuker A, Burnett A, Triller D, Crowther M, Ansell J, Van Cott EM, et al. Reversal of direct oral anticoagulants: Guidance from the Anticoagulation Forum. Am J Hematol. 2019;94(6):697–709. doi: 10.1002/ajh.25475 30916798

[9] Steffel J, Verhamme P, Potpara TS, Albaladejo P, Antz M, Desteghe L, et al. The 2018 European Heart Rhythm Association Practical Guide on the use of non-vitamin K antagonist oral anticoagulants in patients with atrial fibrillation. Eur Heart J. 2018;39(16):1330–93. doi: 10.1093/eurheartj/ehy136 29562325

[10] Kozek-Langenecker SA, Ahmed AB, Afshari A, Albaladejo P, Aldecoa C, Barauskas G, et al. Management of severe perioperative bleeding: guidelines from the European Society of Anaesthesiology: First update 2016. Eur J Anaesthesiol. 2017;34(6):332–95. doi: 10.1097/EJA.0000000000000630 28459785

[11] Spahn DR, Bouillon B, Cerny V, Duranteau J, Filipescu D, Hunt BJ, et al. The European guideline on management of major bleeding and coagulopathy following trauma: fifth edition. Crit Care. 2019;23(1):98. doi: 10.1186/s13054-019-2347-3 30917843 PMC6436241

[12] Gralnek IM, Stanley AJ, Morris AJ, Camus M, Lau J, Lanas A, et al. Endoscopic diagnosis and management of nonvariceal upper gastrointestinal hemorrhage (NVUGIH): European Society of Gastrointestinal Endoscopy (ESGE) Guideline—Update 2021. Endoscopy. 2021;53(3):300–32. doi: 10.1055/a-1369-5274 33567467

[13] Shoamanesh A, Patrice Lindsay M, Castellucci LA, Cayley A, Crowther M, de Wit K, et al. Canadian stroke best practice recommendations: Management of Spontaneous Intracerebral Hemorrhage, 7th Edition Update 2020. Int J Stroke. 2021;16(3):321–41. doi: 10.1177/1747493020968424 33174815

[14] Greenberg SM, Ziai WC, Cordonnier C, Dowlatshahi D, Francis B, Goldstein JN, et al. 2022 Guideline for the Management of Patients With Spontaneous Intracerebral Hemorrhage: A Guideline From the American Heart Association/American Stroke Association. Stroke. 2022;53(7):e282–e361. doi: 10.1161/STR.0000000000000407 35579034

[15] Kalina U, Bickhard H, Schulte S. Biochemical comparison of seven commercially available prothrombin complex concentrates. Int J Clin Pract. 2008;62(10):1614–22. doi: 10.1111/j.1742-1241.2008.01859.x 18691229

[16] CSL Behring. (2018). Kcentra prescribing information [Available from: https://labeling.cslbehring.com/PI/US/Kcentra/EN/Kcentra-prescribing-information.pdf.

[17] Rayatdoost F, Braunschweig T, Maron B, Schochl H, Akman N, Rossaint R, et al. Reversing Rivaroxaban Anticoagulation as Part of a Multimodal Hemostatic Intervention in a Polytrauma Animal Model. Anesthesiology. 2021;135(4):673–85. doi: 10.1097/ALN.0000000000003899 34370811

[18] Herzog E, Kaspereit F, Krege W, Doerr B, Mueller-Cohrs J, Pragst I, et al. Effective reversal of edoxaban-associated bleeding with four-factor prothrombin complex concentrate in a rabbit model of acute hemorrhage. Anesthesiology. 2015;122(2):387–98. doi: 10.1097/ALN.0000000000000520 25419685

[19] Eltringham-Smith LJ, Yu R, Qadri SM, Wang Y, Bhakta V, Pryzdial EL, et al. Prothrombin, alone or in complex concentrates or plasma, reduces bleeding in a mouse model of blood exchange-induced coagulopathy. Sci Rep. 2019;9(1):13029. doi: 10.1038/s41598-019-49552-9 31506556 PMC6736877

[20] Pragst I, Zeitler SH, Doerr B, Kaspereit FJ, Herzog E, Dickneite G, et al. Reversal of dabigatran anticoagulation by prothrombin complex concentrate (Beriplex P/N) in a rabbit model. J Thromb Haemost. 2012;10(9):1841–8. doi: 10.1111/j.1538-7836.2012.04859.x 22812619

[21] Escolar G, Fernandez-Gallego V, Arellano-Rodrigo E, Roquer J, Reverter JC, Sanz VV, et al. Reversal of apixaban induced alterations in hemostasis by different coagulation factor concentrates: significance of studies in vitro with circulating human blood. PLoS One. 2013;8(11):e78696. doi: 10.1371/journal.pone.0078696 24244342 PMC3823858

[22] Perzborn E, Heitmeier S, Laux V, Buchmuller A. Reversal of rivaroxaban-induced anticoagulation with prothrombin complex concentrate, activated prothrombin complex concentrate and recombinant activated factor VII in vitro. Thromb Res. 2014;133(4):671–81. doi: 10.1016/j.thromres.2014.01.017 24529498

[23] Herzog E, Kaspereit F, Krege W, Mueller-Cohrs J, Doerr B, Niebl P, et al. Correlation of coagulation markers and 4F-PCC-mediated reversal of rivaroxaban in a rabbit model of acute bleeding. Thromb Res. 2015;135(3):554–60. doi: 10.1016/j.thromres.2015.01.007 25619440

[24] Schulman S, Gross PL, Ritchie B, Nahirniak S, Lin Y, Lieberman L, et al. Prothrombin Complex Concentrate for Major Bleeding on Factor Xa Inhibitors: A Prospective Cohort Study. Thromb Haemost. 2018;118(5):842–51. doi: 10.1055/s-0038-1636541 29564837

[25] Majeed A, Agren A, Holmstrom M, Bruzelius M, Chaireti R, Odeberg J, et al. Management of rivaroxaban- or apixaban-associated major bleeding with prothrombin complex concentrates: a cohort study. Blood. 2017;130(15):1706–12. doi: 10.1182/blood-2017-05-782060 28835439

[26] Piran S, Khatib R, Schulman S, Majeed A, Holbrook A, Witt DM, et al. Management of direct factor Xa inhibitor-related major bleeding with prothrombin complex concentrate: a meta-analysis. Blood Adv. 2019;3(2):158–67. doi: 10.1182/bloodadvances.2018024133 30658963 PMC6341194

[27] Luo C, Chen F, Chen YH, Zhao CF, Feng CZ, Liu HX, et al. Prothrombin complex concentrates and andexanet for management of direct factor Xa inhibitor related bleeding: a meta-analysis. Eur Rev Med Pharmacol Sci. 2021;25(6):2637–53. doi: 10.26355/eurrev_202103_25428 33829451

[28] Gomez-Outes A, Alcubilla P, Calvo-Rojas G, Terleira-Fernandez AI, Suarez-Gea ML, Lecumberri R, et al. Meta-Analysis of Reversal Agents for Severe Bleeding Associated With Direct Oral Anticoagulants. J Am Coll Cardiol. 2021;77(24):2987–3001. doi: 10.1016/j.jacc.2021.04.061 34140101

[29] Kim PY, Yeh CH, Dale BJ, Leslie BA, Stafford AR, Fredenburgh JC, et al. Mechanistic Basis for the Differential Effects of Rivaroxaban and Apixaban on Global Tests of Coagulation. TH Open. 2018;2(2):e190–e201. doi: 10.1055/s-0038-1649507 31249942 PMC6524873

[30] Chen X, Huang L. Computational model for drug research. Brief Bioinform. 2024;25(3). doi: 10.1093/bib/bbae158 38581423 PMC10998638

[31] Clegg LE, Mac Gabhann F. Molecular mechanism matters: Benefits of mechanistic computational models for drug development. Pharmacol Res. 2015;99:149–54. doi: 10.1016/j.phrs.2015.06.002 26093283 PMC4567444

[32] Collin CB, Gebhardt T, Golebiewski M, Karaderi T, Hillemanns M, Khan FM, et al. Computational Models for Clinical Applications in Personalized Medicine-Guidelines and Recommendations for Data Integration and Model Validation. J Pers Med. 2022;12(2). doi: 10.3390/jpm12020166 35207655 PMC8879572

[33] Hockin MF, Jones KC, Everse SJ, Mann KG. A model for the stoichiometric regulation of blood coagulation. J Biol Chem. 2002;277(21):18322–33. doi: 10.1074/jbc.M201173200 11893748

[34] Chatterjee MS, Denney WS, Jing H, Diamond SL. Systems biology of coagulation initiation: kinetics of thrombin generation in resting and activated human blood. PLoS Comput Biol. 2010;6(9). doi: 10.1371/journal.pcbi.1000950 20941387 PMC2947981

[35] Kogan AE, Kardakov DV, Khanin MA. Analysis of the activated partial thromboplastin time test using mathematical modeling. Thromb Res. 2001;101(4):299–310. doi: 10.1016/s0049-3848(00)00405-9 11248291

[36] Bungay SD, Gentry PA, Gentry RD. A mathematical model of lipid-mediated thrombin generation. Math Med Biol. 2003;20(1):105–29. doi: 10.1093/imammb/20.1.105 12974500

[37] Mann KG, Brummel K, Butenas S. What is all that thrombin for? J Thromb Haemost. 2003;1(7):1504–14. doi: 10.1046/j.1538-7836.2003.00298.x 12871286

[38] Páramo JA. Prothrombin fragments in cardiovascular disease. Adv Clin Chem. 2010;51:1–23. doi: 10.1016/s0065-2423(10)51001-1 20857616

[39] Pabinger I, Brenner B, Kalina U, Knaub S, Nagy A, Ostermann H, et al. Prothrombin complex concentrate (Beriplex P/N) for emergency anticoagulation reversal: a prospective multinational clinical trial. J Thromb Haemost. 2008;6(4):622–31. doi: 10.1111/j.1538-7836.2008.02904.x 18208533

[40] Kintigh J, Monagle P, Ignjatovic V. A review of commercially available thrombin generation assays. Res Pract Thromb Haemost. 2018;2(1):42–8. doi: 10.1002/rth2.12048 30046705 PMC6055498

[41] Giesen PLA, Gulpen AJW, van Oerle R, Ten Cate H, Nagy M, Spronk HMH. Calibrated automated thrombogram II: removing barriers for thrombin generation measurements. Thromb J. 2021;19(1):60. doi: 10.1186/s12959-021-00312-8 34454531 PMC8399793

[42] Ninivaggi M, de Laat-Kremers RMW, Carlo A, de Laat B. ST Genesia reference values of 117 healthy donors measured with STG-BleedScreen, STG-DrugScreen and STG-ThromboScreen reagents. Res Pract Thromb Haemost. 2021;5(1):187–96. doi: 10.1002/rth2.12455 33537543 PMC7845068

[43] Bagot CN, Leishman E. Establishing a reference range for thrombin generation using a standard plasma significantly improves assay precision. Thromb Res. 2015;136(1):139–43. doi: 10.1016/j.thromres.2015.04.020 25956288

[44] Brinkman HJM, Swieringa F, Zuurveld M, Veninga A, Brouns SLN, Heemskerk JWM, et al. Reversing direct factor Xa or thrombin inhibitors: Factor V addition to prothrombin complex concentrate is beneficial in vitro. Res Pract Thromb Haemost. 2022;6(3):e12699. doi: 10.1002/rth2.12699 35494506 PMC9036856

[45] Winter WE, Flax SD, Harris NS. Coagulation Testing in the Core Laboratory. Lab Med. 2017;48(4):295–313. doi: 10.1093/labmed/lmx050 29126301

[46] Gosselin RC, Adcock DM, Bates SM, Douxfils J, Favaloro EJ, Gouin-Thibault I, et al. International Council for Standardization in Haematology (ICSH) Recommendations for Laboratory Measurement of Direct Oral Anticoagulants. Thromb Haemost. 2018;118(3):437–50. doi: 10.1055/s-0038-1627480 29433148

[47] Mueck W, Stampfuss J, Kubitza D, Becka M. Clinical pharmacokinetic and pharmacodynamic profile of rivaroxaban. Clin Pharmacokinet. 2014;53(1):1–16. doi: 10.1007/s40262-013-0100-7 23999929 PMC3889701

[48] Byon W, Garonzik S, Boyd RA, Frost CE. Apixaban: A clinical pharmacokinetic and pharmacodynamic review. Clin Pharmacokinet. 2019;58(10):1265–79. doi: 10.1007/s40262-019-00775-z 31089975 PMC6769096

[49] Skornova I, Samos M, Bolek T, Kamenistakova A, Stanciakova L, Galajda P, et al. Direct Oral Anticoagulants Plasma Levels in Patients with Atrial Fibrillation at the Time of Bleeding: A Pilot Prospective Study. J Cardiovasc Pharmacol. 2021;78(1):e122–e7. doi: 10.1097/FJC.0000000000001038 34173805

[50] Dunois C. Laboratory Monitoring of Direct Oral Anticoagulants (DOACs). Biomedicines. 2021;9(5). doi: 10.3390/biomedicines9050445 33919121 PMC8143174

[51] Ten Cate H, Henskens YM, Lancé MD. Practical guidance on the use of laboratory testing in the management of bleeding in patients receiving direct oral anticoagulants. Vasc Health Risk Manag. 2017;13:457–67. doi: 10.2147/VHRM.S126265 29263674 PMC5732550

[52] Drake TA, Morrissey JH, Edgington TS. Selective cellular expression of tissue factor in human tissues. Implications for disorders of hemostasis and thrombosis. Am J Pathol. 1989;134(5):1087–97. 2719077 PMC1879887

[53] Mackman N. Role of tissue factor in hemostasis, thrombosis, and vascular development. Arterioscler Thromb Vasc Biol. 2004;24(6):1015–22. doi: 10.1161/01.ATV.0000130465.23430.74 15117736

[54] Mandal SK, Pendurthi UR, Rao LV. Cellular localization and trafficking of tissue factor. Blood. 2006;107(12):4746–53. doi: 10.1182/blood-2005-11-4674 16493004 PMC1474814

[55] Connolly SJ, Crowther M, Eikelboom JW, Gibson CM, Curnutte JT, Lawrence JH, et al. Full Study Report of Andexanet Alfa for Bleeding Associated with Factor Xa Inhibitors. N Engl J Med. 2019;380(14):1326–35. doi: 10.1056/NEJMoa1814051 30730782 PMC6699827

[56] Moore EE, Moore HB, Kornblith LZ, Neal MD, Hoffman M, Mutch NJ, et al. Trauma-induced coagulopathy. Nat Rev Dis Primers. 2021;7(1):30. doi: 10.1038/s41572-021-00264-3 33927200 PMC9107773

[57] Toschi V, Lettino M. Inhibitors of propagation of coagulation: factors V and X. Br J Clin Pharmacol. 2011;72(4):563–80. doi: 10.1111/j.1365-2125.2011.04001.x 21545479 PMC3195734

