# Supplementary material for: Modelling the effects of 4-factor prothrombin complex concentrate for the management of factor Xa-associated bleeding
Source: PLoS One. 2024 Sep 27;19(9):e0310883. doi: 10.1371/journal.pone.0310883 (PMC11432878; doi:10.1371/journal.pone.0310883)
Supplement: S1 File — (DOCX) [file pone.0310883.s001.docx]

**Supporting Information**

**S1 Table. Species with non-zero initial values**

| **Species** | **Initial value (M)** | **Species** | **Initial value (M)** |
| --- | --- | --- | --- |
| II | 1.4 x 10^-6^ | XII | 3.4 x10^-7^ |
| TF | 4 x 10^-9^ | C1inh | 2.6 x 10^-6^ |
| VII | 1 x 10^-8^ | XI | 3.1 x 10^-8^ |
| VIIa | 2 x 10^-10^ | a2AP | 1 x 10^-6^ |
| V | 2 x 10^-8^ | a1AT | 4.5 x 10^-5^ |
| Va | 4 x 10^-10^ | PK | 4.5 x10^-7^ |
| VIII | 7 x 10^-10^ | HK | 6.7 x10^-7^ |
| IX | 9 x 10^-8^ | Fbg | 9 x 10^-6^ |
| X | 1.6 x10^-7^ | TM | 1 x 10^-9^ |
| TFPI | 2.5 x 10^-9^ | PC | 6 x 10^-8^ |
| A2M | 3 x 10^-6^ | PS | 3 x 10^-7^ |
| AT | 3.4 x 10^-6^ |  |  |

a, activated; a1AT, alpha-1 antitrypsin; a2AP, alpha 2-antiplasmin; A2M, alpha-2-Macroglobulin; AT, antithrombin; C1inh, C1 inhibitor; Fbg, fibrinogen; HK, High-molecular-weight kininogen; PC, protein C; PK, prekallikrein; PS, protein S; TF, tissue factor; TFPI, tissue factor pathway inhibitor; Tm, thrombomodulin.

**S2 Table. Reactions and kinetic parameters**

| **Reaction** | **ka (M^-1^s^-1^) or (s^-1^)** | **kd (s^-1^)** |
| --- | --- | --- |
| TF + VIIa <-> [TF-VIIa] | 1.3 x 10^8^ | 1 x 10^-3^ |
| TF + VII <-> [TF-VII] | 3.2 x 10^6^ | 3.1 x 10^-3^ |
| [TF-VIIa] + VII -> [TF-VIIa] + VIIa | 4.4 x 10^5^ |  |
| Xa + VII -> VIIa + Xa | 1.3 x10^7^ |  |
| IIa + VII -> IIa + VIIa | 2.3 x 10^4^ |  |
| [TF-VIIa] + X <-> [TF-VIIa-X] | 2.5 x 10^7^ | 1.05 |
| [TF-VIIa-X] -> [TF-VIIa] + Xa | 6 |  |
| [TF-VIIa] + Xa <-> [TF-VIIa-Xa] | 2.2 x 10^7^ | 19 |
| [TF-VIIa] + IX <-> [TF-VIIa-IX] | 1 x 10^7^ | 2.4 |
| [TF-VIIa-IX] -> [TF-VIIa] + IXa | 1.8 |  |
| II + Xa -> IIa + Xa | 7.5 x 10^3^ |  |
| IIa + VIII -> IIa + VIIIa | 2 x 10^7^ |  |
| VIIIa + IXa <-> [VIIIa-IXa] | 1 x 10^7^ | 5 x 10^-3^ |
| [VIIIa-IXa] + X <-> [VIIIa-IXa-X] | 1 x 10^8^ | 1 x 10^-3^ |
| [VIIIa-IXa-X] -> [VIIIa-IXa] + Xa | 8.2 |  |
| IIa + V -> IIa + Va | 2 x 10^7^ |  |
| Xa + Va <-> [Xa-Va] | 4 x 10^8^ | 0.2 |
| [Xa-Va] + II <-> [Xa-Va-II] | 1 x 10^8^ | 100 |
| [Xa-Va-II] -> [Xa-Va-mIIa] | 66 |  |
| [Xa-Va] + mIIa <-> [Xa-Va-mIIa] | 1 x 10^8^ | 66 |
| Xa + TFPI <-> [Xa-TFPI] | 9 x 10^5^ | 3.6 x 10^-4^ |
| [TF-VIIa-Xa] + TFPI <-> [TF-VIIa-Xa-TFPI] | 3.2 x 10^8^ | 1.1 x 10^-4^ |
| [TF-VIIa] + [Xa-TFPI] -> [TF-VIIa-Xa-TFPI] | 5 x 10^7^ |  |
| Xa + AT -> [Xa-AT] | 1.5 x 10^3^ |  |
| mIIa + AT -> [mIIa-AT] | 7.1 x 10^3^ |  |
| IXa + AT -> [IXa-AT] | 490 |  |
| IIa + AT -> [IIa-AT] | 7.1 x 10^3^ |  |
| AT + [TF-VIIa] -> [TF-VIIa-AT] | 230 |  |
| XII -> XIIa | 5 x 10^-4^ |  |
| XII + XIIa <-> [XIIa-XII] | 1 x 10^8^ | 750 |
| [XIIa-XII] -> 2 XIIa | 3.3 x 10^-2^ |  |
| XIIa + C1inh -> [XIIa-C1inh] | 3.6 x 10^3^ |  |
| XIIa + AT -> [XIIa-AT] | 21.6 |  |
| IIa + XI <-> [IIa-XI] | 1 x 10^8^ | 5 |
| [IIa-XI] -> XIa + IIa | 1.3 x 10^-4^ |  |
| XIIa + XI <-> [XI-XIIa] | 1 x 10^8^ | 200 |
| [XI-XIIa] -> XIa + XIIa | 5.7 x 10^-4^ |  |
| XI + XIa <-> [XI-XIa] | 1 x 10^8^ | 200 |
| XIa + AT -> [XIa-AT] | 320 |  |
| XIa + C1inh -> [XIa-C1inh] | 1.8 x 10^3^ |  |
| IXa + X <-> [IXa-X] | 1 x 10^7^ | 6.4 x 10^-2^ |
| [IXa-X] -> Xa + IXa | 7 x 10^-4^ |  |
| Xa + VIII <-> [VIII-Xa] | 1 x 10^8^ | 2.1 |
| [VIII-Xa] -> Xa + VIIIa | 2.3 x 10^-2^ |  |
| VIIa + IX <-> [VIIa-IX] | 1 x 10^8^ | 0.9 |
| [VIIa-IX] -> VIIa + IXa | 3.6 x 10^-5^ |  |
| VIIa + X <-> [VIIa-X] | 1 x 10^8^ | 210 |
| [VIIa-X] -> Xa + VIIa | 1.6 x 10^-6^ |  |
| XIa + a1AT -> [XIa - a1AT] | 100 |  |
| XIa + a2AP -> [XIa - a2AP] | 4.3 x 10^3^ |  |
| XIIa + PK <-> [XIIa - PK] | 1 x 10^8^ | 3.6 x10^3^ |
| [XIIa - PK] -> XIIa + K | 40 |  |
| XII + K <-> [XII - K] | 1 x 10^8^ | 45.3 |
| [XII - K] -> XIIa + K | 5.7 |  |
| PK + K -> K + K | 2.7 x 10^4^ |  |
| K + C1inh -> [K-C1inh] | 1.66 x 10^4^ |  |
| K + A2M -> [K-A2M] | 4.83 x 10^3^ |  |
| K + AT -> [K-AT] | 160 |  |
| [XI-XIa] -> XIa + XIa | 5.7 x 10^-4^ |  |
| K + HK <-> [K-HK] | 1 x 10^8^ | 137 |
| [K-HK] -> K + BK | 0.63 |  |
| BK -> [BK*] | 4.6 x 10^-2^ |  |
| IX + K <-> [IX-K] | 1 x 10^8^ | 15.4 |
| [IX-K] -> IXa + K | 2.22 x 10^-2^ |  |
| IX + XIa <-> [IX-XIa] | 1 x 10^8^ | 29.9 |
| [IX-XIa] -> IXa + XIa | 0.285 |  |
| Fbg + IIa <-> [Fbg-IIa] | 1 x 10^8^ | 636 |
| [Fbg-IIa] -> Fbn1 + IIa + FPA | 84 |  |
| Fbn1 + IIa <-> [Fbn1-IIa] | 1 x 10^8^ | 743 |
| [Fbn1-IIa] -> Fbn2 + IIa + FPB | 7.4 (s^-1^) |  |
| 2 Fbn1 <-> [Fbn1-Fbn1] | 1 x 10^6^ | 6.4 x 10^-2^ |
| [Fbn1-Fbn1] + IIa <-> [Fbn1-Fbn1-IIa] | 1 x 10^8^ | 701 |
| [Fbn1-Fbn1-IIa] -> [Fbn2-Fbn2] + IIa + FPB + FPB | 49 |  |
| Fbn2 + IIa <-> [Fbn2-IIa] | 1 x 10^8^ | 1 x 10^3^ |
| [Xa-Va-mIIa] -> [Xa-Va] + IIa | 15 |  |
| [APC-PS] + VIIIa <-> [APC-PS-VIIIa] | 1 x 10^8^ | 1.6 |
| [APC-PS-VIIIa] -> [APC-PS] + VIIIai | 0.4 |  |
| [APC-PS] + Va <-> [APC-PS-Va] | 1 x 10^8^ | 1.6 |
| [APC-PS-Va] -> [APC-PS] + Vai | 0.4 |  |
| IIa + Tm <-> [IIa-Tm] | 1 x 10^8^ | 0.5 |
| [IIa-Tm] + PC <-> [IIa-Tm-PC] | 1 x 10^8^ | 6.4 |
| [IIa-Tm-PC] -> [IIa-Tm] + APC | 3.6 |  |
| APC + PS <-> [APC-PS] | 1 x 10^8^ | 0.5 |
| V + mIIa <-> [V-mIIa] | 1 x 10^8^ | 6.94 |
| [V-mIIa] -> Va + mIIa | 1.04 |  |
| VIII + mIIa <-> [VIII-mIIa] | 1 x 10^8^ | 13.8 |
| [VIII-mIIa] -> VIIIa + mIIa | 0.9 |  |
| Xa + Rx <-> [Xa-Rx] | 1.7 x 10^7^ | 5 x 10^-3^ |
| [Xa-Va] + Rx <-> [Xa-Va-Rx] | 1.5 x 10^6^ | 6 x 10^-4^ |
| Xa + Ax <-> [Xa-Ax] | 5.7 x 10^6^ | 1.7 x 10^-2^ |
| [Xa-Va] + Ax <-> [Xa-Va-Ax] | 5 x 10^6^ | 2 x 10^-4^ |

Note: irreversible reactions have forward reaction rate units of s^-1^.

a, activated; a1AT, alpha-1 antitrypsin; a2AP, alpha 2-antiplasmin; A2M, alpha-2-Macroglobulin; APC, activated protein C; AT, antithrombin; Ax, apixaban; BK, bradykinin; C1inh, C1 inhibitor; Fbg, fibrinogen; Fbn1, fibrillin-1; Fbn2, fibrillin-2; FPA, fibrinopeptide A; FPB, fibrinopeptide B; HK, High-molecular-weight kininogen; K, kallikrein; mIIa, meizothrombin; PC, protein C; PK, prekallikrein; PS, protein S; Rx, rivaroxaban; TF, tissue factor; TFPI, tissue factor pathway inhibitor; Tm, thrombomodulin.

**S3 Table. Simulations of physiological plasma concentrations**

| **Thrombin generation parameter** | **1 pM TF** | **5 pM TF** |
| --- | --- | --- |
| Peak (nM) | 181 | 198 |
| Lagtime (min) | 1.2 | 2.5 |
| Time to peak (min) | 6.0 | 4.1 |

The peak thrombin levels in the TGA in the presence of a FXa inhibitor (300 ng/mL rivaroxaban) were also simulated using the assay conditions from Figure 1(B) (1). In their *in vitro* data, the FXa inhibitor reduced the peak thrombin to 12% relative to untreated plasma and in our simulation the reduction was 13%.

FXa, activated factor X; TF, tissue factor; TGA, thrombin generation assay.

**S4 Table. Concentrations of components the in silico 4F-PCC product**

| **Species** | **Average amount in 500 IU 4F-PCC*** | **Amount in 25 FIX U/kg^†^** |
| --- | --- | --- |
| II | 590 | 2360 |
| VII | 350 | 1400 |
| IX | 510 | 2040 |
| X | 760 | 3040 |
| PC | 620 | 2480 |
| PS | 460 | 1840 |
| AT | 17 | 68 |

*These are the average values calculated from the range provided in the Product Insert.

^†^4F-PCC doses are based on the FIX levels. All simulations assumed a weight of 80 kg.

4F-PCC, 4-factor prothrombin complex concentrate; AT, antithrombin; FIX, factor IX; IU, international units; PC, protein C; PS, protein S.

**S1 Figure. Effect of TF concentration on simulated PT time**


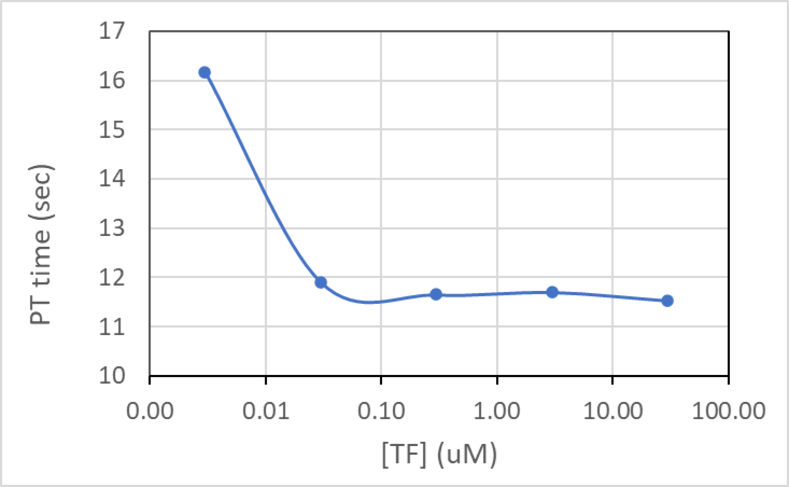


TF, tissue factor; PT prothrombin time.

**S2 Figure. Proportion of rivaroxaban (A) and apixaban (B) bound to FXa**


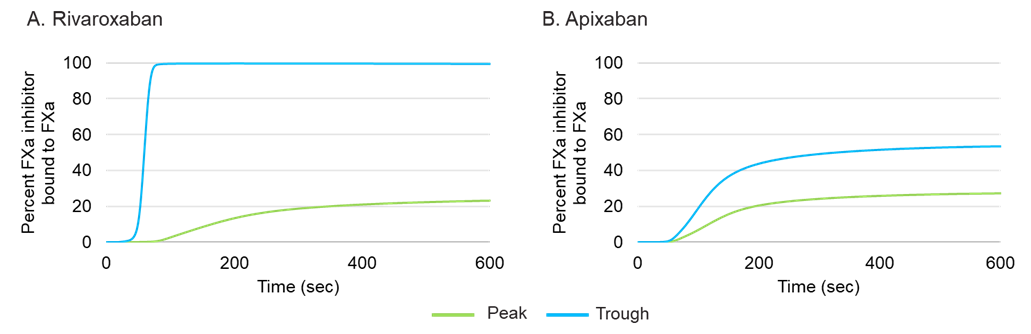
FXa, activated factor X.

**S3 Figure. Effect of inhibitors PC and PS in when simulating individual and binary mixtures of 4F-PCC pro-coagulant components.** Simulation results using average rivaroxaban (A) and apixaban (B) levels with individual and binary mixtures of 4F-PCC components in the presence of the appropriate PC and PS levels (blue squares) and absence of PC and PS (red circles).


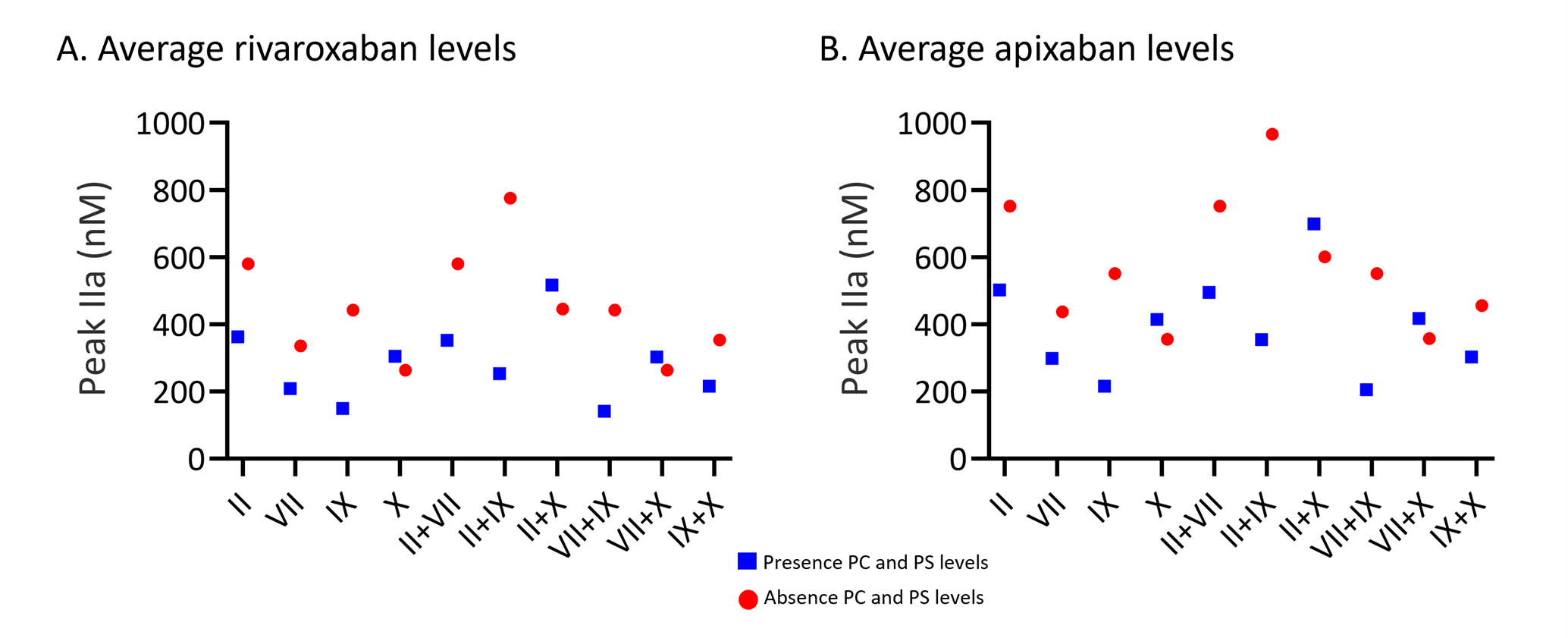


4F-PCC, 4-factor prothrombin complex concentrate; PC, protein C; PS, protein S.

**References**

1. Brinkman HJM, Swieringa F, Zuurveld M, Veninga A, Brouns SLN, Heemskerk JWM, Meijers JCM. Reversing direct factor Xa or thrombin inhibitors: Factor V addition to prothrombin complex concentrate is beneficial in vitro. Res Pract Thromb Haemost. 2022;6(3):e12699.
